# Exploration of risk taking behaviors and perceived susceptibility of colorectal cancer among Malaysian adults: a community based cross-sectional study

**DOI:** 10.1186/1471-2458-13-930

**Published:** 2013-10-07

**Authors:** Sami AR Al-Dubai, Kurubaran Ganasegeran, Aied M Alabsi, Shamsul A Shah, Farid MM Razali, John T Arokiasamy

**Affiliations:** 1 Department of Community Medicine, International Medical University (IMU), No. 126, Jln Jalil Perkasa 19, Bukit Jalil, 57000 Kuala Lumpur, Malaysia; 2 International Medical School, Management and Science University (MSU), University Drive, Off Persiaran Olahraga, Section 13, 40100 Shah Alam, Selangor, Malaysia; 3 Oral Cancer Research And Coordinating Center, Faculty of Dentistry, University of Malaya (UM), Kuala Lumpur 50603 Malaysia; 4 Department of Community Health, UKM Medical Centre, Jalan Yaacob Latif, Bandar Tun Razak, Cheras, 56000 Kuala Lumpur, Malaysia; 5 Perdana University Graduate School of Medicine, Perdana University, Maeps Building, Mardi Complex, 43400 Serdang, SelangorMalaysia

**Keywords:** Behaviors, Colorectal cancer, Health Belief Model, Malaysia, Perceived susceptibility

## Abstract

**Background:**

Perceived susceptibility to an illness has been shown to affect Health-risk behavior. The objective of the present study was to determine the risk taking behaviors and the demographic predictors of perceived susceptibility to colorectal cancer in a population-based sample.

**Methods:**

A cross-sectional study was carried out among 305 Malaysian adults in six major districts, selected from urban, semi-urban, and rural settings in one state in Malaysia. A self-administered questionnaire was used in this study. It was comprised of socio-demographics, risk-taking behaviors, and validated domains of the Health Belief Model (HBM).

**Results:**

The mean (± SD) age of the respondents was 34.5 (± 9.6) and the majority (59.0%) of them were 30 years or older. Almost 20.7% of the respondents felt they were susceptible to colorectal cancer. Self-reported perceived susceptibility mirrored unsatisfactory screening behaviors owing to the lack of doctors’ recommendation, ignorance of screening modalities, procrastination, and the perception that screening was unnecessary. Factors significantly associated with perceived susceptibility to colorectal cancer were gender (OR = 1.8, 95% CI 1.0-3.3), age (OR = 2. 2, 95% CI 1.2-4.0), ethnicity (OR = 0. 3, 95% CI 0.2-0.6), family history of colorectal cancer (OR = 3. 2, 95% CI 1.4-7.4) and alcohol intake (OR = 3.9, 95% CI 2.1-7.5).

**Conclusion:**

The present study revealed that screening behavior among respondents was unsatisfactory. Hence, awareness of the importance of screening to prevent colorectal cancers is imperative.

## Background

Colorectal cancer (CRC) is one of the most common malignancies among adults in both developed and developing countries [1]. Currently, it is the third most commonly diagnosed malignancy and the fourth leading cause of death worldwide [2], with more than 940,000 cases diagnosed annually [3]. Recent reports indicate that it is the fastest emerging gastrointestinal tract cancer in Asia Pacific [4]. In Malaysia, CRC is the most common malignancy in men and the third commonest malignancy in women (after breast and cervical cancer) [5].

Colorectal cancer is characterized as developing over long periods of time and is a localized and curable condition [6]. The survival rate for localized disease is 90%, compared to less than 10% for metastatic disease [7]. Screening for CRC has been shown to reduce mortality as it facilitates early detection and prevention of the malignancy [8-10]. The recommended screening methods for early detection of colorectal cancer are fecal occult blood test (FOBT), flexible sigmoidoscopy [11], barium enema and colonoscopy [12].

Risk taking behaviors are associated with perceived susceptibility for cancer [13]. Individuals engaged in detrimental health behaviors would perceive a higher susceptibility to malignancies [14]. Smokers were found to perceive a higher susceptibility of developing cancer than non-smokers [15,16]. In a meta-analysis study, a significant association was found between perceived a susceptibility of colorectal cancer and the consumption of fruits and vegetable [17]. Alcohol consumption, obesity and low levels of activity were also reported as risk factors of CRC [18-20].

Social cognitive frameworks in preventive health, namely the Health Belief Model (HBM) [21], the Protection Motivation Theory [22], and the Precaution Adoption Process Model [23] view risk perceptions with perceived susceptibility as a catalyst for primary prevention [24]. Perceived susceptibility advocates the “motivational engine” for precautionary behaviors [13]. However, social cognitive critics debate the validity of variables from these models, which tend to predict behavioral intention rather than the behavior itself [25]. McQueen et al., (2010) posed the alternate hypothesis that perceived susceptibility advocates change in psychosocial predictors influencing subsequent intentions and behavior [26]. The HBM relates a socio-psychological theory of decision making to the individual health-related behaviors and it included four dimensions: Susceptibility, Severity, Benefits and Costs [21]. This study believes that understanding the determinants of perceived susceptibility to colorectal cancer is a crucial element in constructing cancer preventive health behaviors.

This study aims to explore risk taking behaviors and perceived susceptibility to colorectal cancer among healthy communities from different geographical settings (urban, semi-urban and rural) and ages. This study will overcome gaps and weaknesses of previous studies by expanding its socio-demographic factors and other relevant covariates concerning colorectal cancer susceptibility and screening behaviors by using the validated Health Belief Model (HBM) [21] among the Malaysian population.

## Methods

### Study setting and population

A community-based cross-sectional study was conducted in three different geographical settings (urban, semi-urban, and rural areas) within the state of Selangor, Malaysia. This state includes six major districts, namely Subang Jaya, Petaling Jaya, Selayang, Klang, Kuala Selangor and Kuala Langat. Subang Jaya and Petaling Jaya are urban settings, Selayang and Klang are semi-urban, and Kuala Selangor and Kuala Langat are rural. The sample size required for this study was estimated to be 280 respondents. This calculation was based on previous study parameters [27] using Lwanga and Lemeshow’s equation (1991). We added 10% of the calculated sample size (28) to compensate for missing data or non-response and so the sample size rose to 308. Respondents aged ≥18 years, and who lived for at least one year in these districts, were also included in this study. Respondents were approached at various commercial settings and major streets within the selected residential areas using convenience sampling. Questionnaires were administered in both English and the local language.

### Ethical consideration

The purpose of the study was explained to respondents. Confidentiality and their right to withdraw were ensured. Participants received a written description of the purpose and aims of the study along with the study questionnaires and participant consent was obtained. Approval was obtained from the ethics committee of the International Medical School in the Management and Science University (MSU), Malaysia.

### Study instruments

A self-administered questionnaire was developed using previous literatures and the validated Health Belief Model Scale (HBM) for colorectal cancer [21]. Socio-demographics included 10 questions relating to gender, age, race, residency area, marital status, level of education, occupation, family income, family history of any cancer, and family history of colorectal cancer. We designed a five-item mini-subscale to assess the risk taking behaviors of colorectal cancer (smoking, alcohol intake, fruits and vegetable consumption, exercise and obesity). The Body Mass Index (BMI) was calculated as weight in kilograms divided by height in square meters (kg/m2). This study adopted the World Health Organisation’s (WHO) BMI cut-off points for Asian populations. A BMI <18.5 kg/m^2^ was categorized as underweight, 18 · 5–22 · 9 kg/m^2^ as normal and ≥ 23.0 kg/m^2^ as overweight. The latter was further classified as pre-obese (23.0–27.4 kg/m^2^), obese Class I (27.5–34.9 kg/m^2^), obese Class II (35.0–39.9 kg/m^2^), and obese Class III (≥ 40 kg/m^2^) [28]. To ease analysis “underweight” and “normal range” were categorized as 'normal’ while “pre-obese” and “obese Class I, II and III” were categorized as 'obese’. The validated HBM was used in this study. It assessed four main constructs namely: perceived susceptibility (1 item), perceived severity (1 item), perceived benefits (1 item) and perceived barriers (17 items) [21]. Response options include “Agree” or “Disagree”. the questionnaire was pilot-tested among 10 respondents.

### Statistical analyses

Data analysis was performed using the Statistical Package of Social Sciences (SPSS) software, version 16.0. Descriptive statistics were obtained for all variables in the study. The chi-square test was used to assess the association between perceived susceptibility and categorical variables in this study. For variables with three or more categories, simple logistic regression analysis was used to obtain the odds ratio (OR). Multiple logistic regression analysis using the Backward Wald technique was performed to obtain factors associated with perceived susceptibility to colorectal cancer. All independent variables that had significant associations with perceived susceptibility in bivariate analysis were included in the multivariate analysis. Multi-collinearity between independent variables was checked for by the values of standard errors (SE). The accepted level of significance in this study was set below 0.05 (p < 0.05).

## Results

### Socio-demographic characteristics of the respondents

The data of 305 respondents were included in the analysis (three questionnaires were excluded due to missing data). The mean (±SD) age of respondents was 34.5 (±9.6) years with the majority aged 30 years or older (59.0%). One hundred eighty five were Malays (60.7%), 175 were married (57.7%), 154 were tertiary educated (50.5%), 278 were currently employed (91.5%), 26 respondents (8.5%) had a family history of colorectal cancer and 32 (10.5%) had a family history of other malignancies (Table 1).

**Table 1:** Socio -demographic characteristics of respondents (n = 305)

**Characteristics**	**N**	**%**
**Gender**		
Male	185	60.7
Female	120	39.3
**Age**		
≤ 30 years	125	41.0
> 30 years	180	59.0
**Race**		
Malay	185	60.7
Chinese	74	24.3
Indian	46	15.0
**Residency**		
Urban	105	34.4
Semi-urban	100	32.8
Rural	100	32.8
**Marital status**		
Married	176	57.7
Unmarried	129	42.3
**Education level**		
High school or less	151	49.5
College/University	154	50.5
**Occupation**		
Employed	279	91.5
Unemployed	26	8.5
Family income (MYR) /month*		
< 3000	178	58.3
3000-5000	85	27.9
> 5000	42	13.8
**Family history of any cancer**		
Yes	32	10.5
No	273	89.5
**Family history of colorectal cancer**		
Yes	26	8.5
No	279	91.5

* 1USD = MYR3 at the time of study.

### Risk taking behaviors of colorectal cancer among respondents

Table 2 exhibits the respondents’ risk taking behaviors regarding colorectal cancer. One hundred eighty three were obese (60.0%), 75 (24.6%) consumed fruits and vegetables less than three times per week, 74 (24.3%) and 51 (16.7%) respondents respectively reported cigarette smoking and drinking alcohol, while 264 (13.4%) reported performing exercise less than three times per week.

**Table 2:** Risk taking behaviors of colorectal cancer among respondents (n = 305)

**Risky behaviors**	**N**	**%**
**Smoking**		
Yes	74	24.3
No	231	75.7
**Drinking alcohol**		
Yes	51	16.7
No	254	83.3
**Exercise**		
< 3 times per week	41	13.4
≥ 3 times per week	264	86.6
**Consuming fruits & vegetable**		
< 3 times per week	75	24.6
≥ 3 times per week	230	75.4
**Obesity**		
Obese	183	60.0
Normal	122	40.0

### Perception of colorectal cancer and screening according to Health Belief Model

Two hundred and twenty two of the respondents (79.3%) believed that the risk of getting colorectal cancer was low, 231 (75.7%) believed that colorectal cancer is serious if diagnosed late and 237 (77.7%) of respondents were not worried about discovering colorectal cancer if they had screening tests. In addition, 169 participants (55.4%) reported that they did not have an FOBT because it was “not recommended by a doctor”. Similarly, 153 (50.2%) respondents reported the same reason for not having a flexible sigmoidoscopy. Other stated reasons are shown in Table 3.

**Table 3:** Perception of colorectal cancer and screening among respondents (n = 305)

**Statement**	**Agree**	**Disagree**
	**N (%)**	**N (%)**
**Perceived susceptibility**		
My chance of getting colorectal cancer is great	63 (20.7)	242 (79.3)
**Perceived severity**		
Colorectal cancer may be serious if it is found late	231 (75.7)	74 (24.3)
**Perceived benefits**		
If I have colorectal cancer, I will have a good chance of survival if the cancer is found early	204 (66.9)	101 (33.1)
**Perceived barriers**		
**Reasons for not getting FOBT**		
Procrastination	113 (37.0)	192 (63.0)
Didn’t know I should have it	120 (39.3)	185 (60.7)
Do not think it’s necessary	71 (23.3)	234 (76.7)
Not recommended by my doctor	169 (55.4)	136 (44.6)
Too embarrassing	55 (18.0)	250 (82.0)
I don’t have health problems	127 (41.6)	178 (58.4)
**Concerns about FOBT**		
Worried that FOBT is messy	75 (24.6)	230 (75.4)
Worried that FOBT is inconvenient	95 (31.1)	210 (68.9)
**Reasons for not getting FlexibleSigmoidoscopy**		
Procrastination	115 (37.7)	190 (62.3)
I don’t know I should have it	118 (38.7)	187 (61.3)
I don’t think it’s necessary	87 (28.5)	218 (71.5)
It was not recommended by my doctor	153 (50.2)	152 (49.8)
I have no symptoms	132 (43.3)	173 (56.7)
It is painful	82 (26.9)	223 (73.1)
**Concerns about Flexible Sigmoidoscopy**		
Worried that Flexible Sigmoidoscopy is embarrassing	96 (31.5)	209 (68.5)
Worried that Flexible Sigmoidoscopy is painful	91 (29.8)	214 (70.2)
**Perceived barriers to CRC screening**		
Fear of discovering cancer	68 (22.3)	237 (77.7)

### Perceived susceptibility to colorectal cancer by socio-demographic characteristics

Table 4 shows the association between socio-demographic factors and perceived susceptibility to colorectal cancer. Perceived susceptibility was higher in men compared to women (OR = 1.8, 95% CI 1.0-3.3, p = 0.045), and in those aged >30 years compared to younger respondents (OR = 2.2, 95% CI 1.2-4.0, p = 0.011). Perceived susceptibility was lower among Malays and Indians when compared with Chinese (OR = 0.3, 95% CI 0.2-0.6, p = 0.001), (OR = 0.3, 95% CI 0.1-0.7, p = 0.007 respectively). Respondents with a family history of colorectal cancer perceived a higher susceptibility in comparison to those without (OR = 3.2, 95% CI 1.4-7.4, p = 0.004).

**Table 4:** Association between perceived susceptibility of colorectal cancer and demographic variables (n = 305)

**Characteristics**	**Perceived higher chance of having colorectal cancer**	**Perceived lower chance of having colorectal cancer**	**OR**	**95% CI**	**P-value**
	**N (%)**	**N (%)**			
**Gender**					
Male	45 (24.3)	140 (75.7)	1.8	1.0-3.3	0.045
Female	18 (15.0)	102 (85.0)	1		
**Age**					
≤ 30 years	17 (13.6)	108 (86.4)	1		
> 30 years	46 (25.6)	134 (74.4)	2.2	1.2-4.0	0.011
Race*					
Malay	30 (16.2)	155 (83.8)	0.3	0.2-0.6	0.001
Indian	6 (13.0)	40 (87.0)	0.3	0.1-0.7	0.007
Chinese	27 (36.5)	47 (63.5)	1		
Residency*					
Urban	29 (27.6)	76 (72.4)	1		
Semi-urban	17 (17.0)	83 (83.0)	0.5	0.3-1.1	0.071
Rural	17 (17.0)	83 (83.0)	0.5	0.3-1.1	0.071
**Marital status**					
Married	42 (23.9)	134 (76.1)	1.6	0.9-2.9	0.106
Unmarried	21 (16.3)	108 (83.7)	1		
**Education level**					
High school or less	30 (47.6)	124 (51.2)	1		
College/University	33 (52.4)	118 (48.8)	1.2	0.7-2.0	0.609
**Occupation**					
Employed	57 (20.4)	222 (79.6)	1		
Unemployed	6 (23.1)	20 (76.9)	1.2	0.4-3.0	0.750
Family income (MYR)*					
< 3000	33 (18.5)	145 (81.5)	1		
3000-5000	21 (24.7)	64 (75.3)	1.4	0.8-2.7	0.248
> 5000	9 (21.4)	33 (78.6)	1.2	0.5-2.7	0.669
**Family history of any cancer**					
Yes	53 (19.4)	220 (80.6)	1		
No	10 (31.2)	22 (68.8)	1.9	0.8-4.2	0.122
**Family history of colorectal cancer**					
Yes	11 (42.3)	15 (57.7)	3.2	1.4-7.4	0.004
No	52 (18.6)	227 (81.4)	1		

* Simple logistic regression was used to obtain the OR.

### Association between risk taking behaviors and perceived susceptibility to colorectal cancer

Table 5 shows the association between risk taking behaviors and perceived susceptibility to colorectal cancer. Alcohol consumption was the only risk taking behavior that had a significant association with perceived susceptibility (OR = 3.9, 95% CI 2.1-7.5 p < 0.001).

**Table 5:** Association between risk taking behaviors and perceived susceptibility among respondents (n = 305)

**Characteristics**	**Believe chance of having colorectal cancer is high**		OR*	**95% CI**	**P-value**
	**Yes**	**No**			
	**N (%)**	**N (%)**			
**Smoking**					
Yes	18 (28.6)	56 (23.1)	1.3	0.7-2.5	0.370
No	45 (71.4)	186 (76.9)			
**Alcohol**					
Yes	22 (34.9)	29 (12.0)	3.9	2.1-7.5	<0.001
No	41 (65.1)	213 (88.0)			
**Exercise**					
Less than three times per week	7 (11.1)	34 (14.0)	0.8	0.3-1.8	0.542
Three or more times per week	56 (88.9)	208 (86.0)			
**Consume fruits and vegetable**					
Less than three times per week	17 (27.0)	58 (24.0)	1.2	0.6-2.2	0.620
Three or more times per week	46 (73.0)	184 (76.0)			
**Body Mass Index (BMI)**					
Obese	28 (44.4)	94 (38.8)	1.3	0.7-2.2	0.419
Normal	35 (55.6)	148 (61.2)			

### Factors associated with perceived susceptibility among respondents in multiple logistic regression analysis

In the multiple logistic regression analysis, age and race were significantly associated with perceived susceptibility to colorectal cancer. Respondents aged > 30 years were more likely to report that they were susceptible to colorectal cancer compared to those aged ≤ 30 years (OR = 2.6, 95% CI 1.4-4.9). A Malay (OR = 95% CI 0.2-0.6) or Indian (OR = 0.2, 95% CI 0.1-0.7) was less likely to report that they were susceptible to cancer compared to Chinese (Table 6).

**Table 6:** Multiple logistic regression analysis (Backward Wald); predictors of perceived susceptibility among respondents (n = 305)

**Predictors**	**B**	**SE**	**Wald**	**Exp(B)**	**95% CI**	**P value**
**Age**						
≤ 30 years	Ref	Ref	Ref	Ref	Ref	Ref
> 30 years	0.940	0.327	8.269	2.6	1.4-4.9	0.004
**Race**						
Malay	-1.236	0.325	14.430	0.3	0.2-0.6	< 0.001
Indian	-1.419	0.509	7.782	0.2	0.1-0.7	0.005
Chinese	Ref	Ref	Ref	Ref	Ref	Ref

Variables entered: Gender, age, race, residency, marital status, family history of any cancer and family history of colorectal cancer. Exp(B) gives the Odds Ratio.

## Discussion

This study aimed to explore risk taking behaviors and determine potential factors affecting perceived susceptibility to colorectal cancer among the Malaysian population. Of the 305 respondents surveyed, 20.7% perceived high chances of having colorectal cancer. In the final model, age and race were significantly associated with perceived susceptibility to colorectal cancer.

To the best of our knowledge, this study was the first Malaysian study to assess self-reported risk-taking behaviour and perceived susceptibility to colorectal cancer. The estimated rate of perceived susceptibility reported in the present study was comparatively higher to that found in a British sample (17%) [14] but relatively lower than that reported from the United States of America (USA)(29-50%) [29-31].

Health behavioral theories predicted higher intentions of preventive actions among individuals with greater susceptibility to a disease [13,32]. However, in the present study, self-reported susceptibility mirrored unsatisfactory preventive behaviours. The barriers to screening for colorectal cancer in this study were consistent with previous studies from Malaysia on the barriers of CRC screening [33] and USA [8,34].

In the present study, perceived susceptibility to colorectal cancer was significantly higher among men. A previous study by Wardle et al., (2005) [35] found that men had low perceived susceptibility while some studies found no relationship between gender and perceived susceptibility [36-38]. Our study’s findings on the association between perceived susceptibility and age was consistent with previous studies [14,39].

A new finding in this study was the significant association between perceived susceptibility and race. Despite the high incidence of colorectal cancer among ethnic Chinese in Malaysia [33], this study found higher perceived susceptibility among ethnic Malays and Indians in comparison to Chinese. A possible explanation could be due to a lack of information among respondents. In the literature, the association between ethnicity and perceived susceptibility was masked. A study by Shokar et al., (1990) concluded that whites were more likely to contract colorectal cancer than blacks.

This study found a significant relationship between perceived susceptibility and family history of colorectal cancer which was consistent with previous studies [13,29,31,40].

This study also found a significant association between alcohol intake and the perceived susceptibility to colorectal cancer which was, again, consistent with a previous study [41]. Our finding of the relationship between smoking and perceived susceptibility was inconsistent with some previous studies [15,16]. Although some longitudinal studies had observed a positive relationship between perceived risk and subsequent behaviour, that association was weak and unsatisfactory. Some studies, however, find no association or even a negative one [32,42].

The cross-sectional design of the current study can test the “accuracy hypothesis” that asserts that perceptions of risk at a certain time properly reflect one’s risk behaviours at that time [41]. Thus, this design can be useful for identifying information deficits and the areas where further education is needed. The failure of the current study to find an association between the perceived susceptibility and risk taking behaviors could be attributed to the natural causal relationship between those variables in which the cross-sectional design was not appropriate [41]. A second possible reason could be due to an inadequate specification of the links between perceived susceptibility and risk taking behaviour in the study questionnaire. In other words, our respondents may not answer the “perceived susceptibility” questions according to their awareness that smoking is a risk for developing cancer. To avoid such bias in the future, this study recommends phrasing the question in another way, for example, “If you don’t change your smoking behaviour, what is your chance of getting colorectal cancer in the future?” By using such a question, we can link the perceived susceptibility to the behaviour. An alternative way is to assess the relationship between the perceived susceptibility and the intention to quit or stop the risky behaviours. Although intentions may not necessarily predict or reflect the actual behaviours, it could be considered as an intermediate step towards action.

The cross-sectional nature of this study could not establish the causal relationships between perceived susceptibility and behaviour. Thus, further exploration is required through prospective and meta-analysis studies.

## Conclusions

This study found unsatisfactory screening behaviors due to the lack of doctors’ recommendation, unawareness of screening modalities, procrastination, and a negative apprehension that screening was unnecessary. Perceived susceptibility was associated significantly with age, race, and alcohol intake. Community health education and a health promotion program of colorectal cancer risk factors and screening methods should be conducted among the community. Doctors and health care providers should be engaged in public education.

## Competing interests

The authors declare that they do not have competing interests.

## Authors’ contributions

SAR and KG designed the research protocol. MFMR conducted data collection, data cleaning and normality check. SAR, SAS and AMA conducted data analyses and interpretation. KG, SAR and MFMR wrote the paper. SAS, JTA and, SAR revised the final draft critically for important intellectual content. All authors read and approved the final manuscript.

## Pre-publication history

The pre-publication history for this paper can be accessed here:

http://www.biomedcentral.com/1471-2458/13/930/prepub
